# Predicting and Comparing Students’ Online and Offline Academic Performance Using Machine Learning Algorithms

**DOI:** 10.3390/bs13040289

**Published:** 2023-03-28

**Authors:** Barnabás Holicza, Attila Kiss

**Affiliations:** 1 Department of Information Systems, ELTE Eötvös Loránd University, 1117 Budapest, Hungary; 2 Department of Informatics, János Selye University, 945 01 Komárno, Slovakia

**Keywords:** students’ performance, e-learning, machine learning, k-nearest neighbors, decision tree, random forest, support vector machine

## Abstract

Due to COVID-19, the researching of educational data and the improvement of related systems have become increasingly important in recent years. Educational institutions seek more information about their students to find ways to utilize their talents and address their weaknesses. With the emergence of e-learning, researchers and programmers aim to find ways to maintain students’ attention and improve their chances of achieving a higher grade point average (GPA) to gain admission to their desired colleges. In this paper, we predict, test, and provide reasons for declining student performance using various machine learning algorithms, including support vector machine with different kernels, decision tree, random forest, and k-nearest neighbors algorithms. Additionally, we compare two databases, one with data related to online learning and another with data on relevant offline learning properties, to compare predicted weaknesses with metrics such as F1 score and accuracy. However, before applying the algorithms, the databases need normalization to meet the prediction format. Ultimately, we find that success in school is related to habits such as sleep, study time, and screen time. More details regarding the results are provided in this paper.

## 1. Introduction

In a sustainable country, the quality of education plays a critical role in supporting global sustainable development [1]. Investing in education, according to the World Bank, can lead to economic growth and poverty reduction [2]. However, challenges related to the internet and social factors often cause many students to struggle across different fields [3]. Technological advancements now enable instructors to use data mining and analytical approaches to analyze vast databases for patterns that represent their students’ behavior and learning [4]. The COVID-19 pandemic has significantly altered the way education is delivered, with e-learning emerging as a viable option for many institutions [5]. However, this has also presented various challenges to students, such as attention deficit disorder and loneliness [6]. Educational data created through different platforms, including e-learning, e-admission systems, and automated result management systems, can be analyzed using educational data mining approaches to gain insights into student performance [7]. Deep learning techniques have been used to automatically extract high-level features from unprocessed data, leading to better performance on difficult tasks [8]. One essential application of educational data mining is predicting student performance based on prior academic data. To achieve this, an automated method for forecasting student performance is needed [7]. Researchers have used various indicators/variables to predict student performance across different fields, including engineering and medicine, at various levels of education [9,10].

In this context, machine learning (ML) algorithms can potentially improve academic performance. By analyzing the performance data of individual students, ML algorithms can identify their strengths and weaknesses and provide personalized recommendations to improve academic performance. For example, an ML algorithm can analyze a student’s past exam results, homework assignments, and class participation data to identify areas where they may need more support. The algorithm can then provide targeted learning resources or suggest specific areas of focus to help the student improve. Moreover, ML algorithms can enable adaptive testing, where the algorithm adjusts the difficulty level of questions based on the student’s performance. This approach can ensure that students are appropriately challenged, leading to a better assessment of their understanding and ultimately improving their academic performance. Additionally, ML algorithms can be used to analyze large volumes of educational data, such as student records and demographic information, to identify patterns and insights that can inform educational policies and practices. For example, an ML algorithm can analyze the performance data of a school’s student population and identify areas where more support may be needed. This information can then be used to inform targeted interventions, such as tutoring or mentoring programs, to help students who may be struggling.

## 2. Related Works

### 2.1. The New Way of Teaching

Online learning has been found to be beneficial and convenient for students’ interactions. However, it cannot fully replace conventional learning methods due to challenges with infrastructure, engagement strategies, semantic web strategies, and the need for improved knowledge management. Machine learning techniques have been proposed to address these issues, as they perform similarly to traditional models, but with longer run times and more precise hyperparameters. Educational data from various platforms, such as e-learning, e-admission systems, and automated result management systems, can be analyzed through educational data mining approaches to gain insights into student performance. An attention-based *bidirectional long short-term memory (BiLSTM)* [11] network has been proposed to predict student performance by analyzing their historical data, such as previous grades and exam points. The model includes an embedding layer to generate a feature matrix, a BILSTM Layer with two hidden layers, an attention layer to focus on important words, and an output layer that predicts student grades using the sigmoid function. However, this model only considers significant features like grades, and could be further developed by incorporating students’ social attributes. One potential evaluation tool for online learning is the *SWOT (strengths weaknesses opportunities threats) analysis* [12], which is commonly used in marketing but can also be applied to other fields. By comparing strengths, weaknesses, opportunities, and threats, the effectiveness of online learning can be assessed. Online learning is a new way of teaching and studying, facilitated by technological devices like smartphones.

### 2.2. Behavior Studies and the Application of Markov Models

In predicting students’ performance, cognitive models play a very important role in recent studies. Using *deep cognitive diagnostic systems (deepCFD: deep computational fluid dynamics )* [13], a better accuracy rate can be achieved, and this system can predict students’ scores on both objective and subjective problems. Cognitive diagnosis models are used to understand a student’s cognitive abilities by examining their mastery of specific skills. These models distinguish between compensatory and non-compensatory relationships among skills. Data mining methods often use matrix factorization to infer a student’s hidden properties and improve efficiency through pruning strategies. DeepCFD integrates cognitive diagnosis models and deep learning methods to understand a student’s cognitive abilities. It models a student’s skill mastery and problem proficiency based on their problem-solving responses, capturing the importance of skills to problem-solving. A worrying aspect of online course continuation is dropout. However, the high dropout rate and the difficulty in making an accurate prognosis continue to be problems for online education systems. Researchers have realized that the problem of dropout prediction is essentially one of sequence labeling or time series prediction. For this reason, they have presented [14] two models: the input–output hidden Markov model and the logistic regression with a regularization term (IOHMM). The model called transition probabilities processes sequential input of discrete times and can classify, produce, or predict tasks. Logistic regression models are learned independently for each student for each week, based on features extracted from their online learning system logs.

Students’ achievements can reflect their performance by looking at the students’ course mastery and lecturers’ teaching level. It not only serves as a form of early warning and prompt correction for students and teachers, but it also gives university decision-makers a way to assess the caliber of courses. Using an *evolutionary spiking neural network model* [15] to predict was found to be beneficial in this area of studies. The model uses an evolutionary algorithm to optimize the hyperparameters in order to obtain the best output performance. In e-learning systems, quality can be assured by maximizing student’s performances. These measures can be predicted, and can be a sign of change which should be implemented for future improvements. These predictions can be calculated by machine learning algorithms using e-learning behavior data. These models can generate real-time supervision and feedback during the learning process. A *behavior classification-based e-learning performance (BCEP) prediction framework* [10] was proposed, which uses behavioral data as feature fusion to obtain a classification model to obtain the category feature values. The BCEP prediction framework consists of data cleaning, behavior classification, feature fusion, and model training. It achieves more accurate predictions of student achievement with reduced computational complexity and increased mobility and versatility. The paper also introduces the PBC model, which categorizes e-learning behaviors into four categories and improves performance prediction compared to existing methods.

Researchers have also experimented with *eye-tracking technology* [16] to analyze and predict students’ learning performance during embodied-based activities. They used different machine learning algorithms to combine gaze features into students’ learning profiles. Their predictive model incorporates learning human body anatomy and uses multimodal data. Using their generated dataset, the accuracy of the support vector in predicting student performance increased when incorporating eye-gaze features. Most earlier studies on schooling have used the cross-sectional study design, which involves comparing the results of the pretest and post-test. However, a particular study demonstrates how time series analysis using a *hidden Markov model (HMM)* [17] can be used to evaluate students’ knowledge during the engagement. HMM is a widely used method for modeling time series data. It has rich mathematical structure and practical algorithms for computing model components. The Baum–Welch or EM algorithm can be used to estimate HMM parameters when the time series is not labeled and the mapping between observations and states is unknown. In continuous observation cases (CHMM), emission probabilities are used instead. In this study, a game was developed, and students could learn classes through the game. Based on their behaviors, researchers could predict the students’ post-test results. To increase engagement, gamification was used, which involves integrating game concepts into nongame environments, such as a website, online community, learning management system, or company intranet. Gamification aims to engage customers, staff members, and partners to encourage cooperation, sharing, and interaction. The researchers used *machine learning algorithms* [18] to provide effective continuous feedback to students based on their academic results during the course. The students were clustered into different groups based on experience points (XP) that they received during the gamified course. Balancing was achieved using virtual students, and then prediction was generated by their final grades using different algorithms such as expectation maximization (EM).

### 2.3. On-Site Research

A study was conducted [19] with university students from various Chinese provinces who had experience learning online to determine the relationship between behavioral intention (BI) to adopt online learning, perceived usefulness (PU), perceived ease of use (PEU), self-regulated online learning (SR), and online learning self-efficacy (SE). The gathered data were examined using *structural equation modelling (SEM)*. Covariance-based SEM was chosen to identify causal relationships among factors based on specific conditions. Nonstandardized coefficients, standard errors, standardized coefficients, critical ratios, and significance levels were calculated to test hypotheses, with gender, nationality, and grade as control variables. According to the findings, PU significantly improves BI, while SR significantly improves PEU, PU, and BI. This study demonstrates how students’ online habits, such as utilizing their phones and taking notes, have a significant impact on their performance metrics.

A study [20] examined the academic performance of Chilean schoolchildren from the province of Biobio to determine the relationship between perceptions of cognitive processes, such as memory, processing speed, attention, execution of complex tasks, and anxiousness. It was done using a cross-sectional analytical design. According to the findings, 20.3% of the pupils felt extremely anxious, while 16.8% felt distracted. Differences in grades and GPAs were observed across all of the assessed disciplines depending on how cognitive processes were perceived.

While a student’s grades (or GPA) can be the main factor of declining performance, other factors can influence prediction accuracy and scores, such as backgrounds, selected courses, and courses that are not equally informative or impactful on the student’s grades. A proposed method [21] utilizes other factors for prediction through two approaches: a bilayered structure and a data-driven approach based on latent factor models and probabilistic matrix factorization. It is also worth noting that numerous factors, other than the educational status of the students (like grades, course material, teaching quality), can influence students’ academic performances [22], such as socioeconomic factors, e.g., family income and parental level of education, which are not considered in the predictions.

### 2.4. Previous Applications of Decision Trees and Artificial Neural Networks

In some chosen publications, *decision tree (DT) and ensemble learning models* [23] have been used. Neural networks (NNs) or transfer learning with the appropriate layers can be used to make an objective choice about the model that is best for the data obtained. The only issue with these models is that they are often biased towards certain measures. In fact, a wide range of metrics that are suitable can be chosen to gauge performance, while also selecting the best approach method: classification or regression. The most important factor in this method is that researchers are provided with a large amount of student data, and changes in the data can be analyzed for further advancement. An approach that yielded results close to the data mining approaches was the *artificial neural networks (ANNs)* [24], with very high prediction accuracy. The sample size, level, field of education, or context of the study did not appear to have any effect on the methodology’s degree of accuracy. In addition to some demographic factors, such as gender, student scores, such as cumulative GPA, were widely employed as input variables due to the simplicity and availability of the data. For prediction, a large amount of training data is needed, and its features must also be balanced. This collection of quantitative and qualitative data from many sources such as exam centers, virtual courses, e-learning educational systems is not a simple task to do. After gathering, difficulties can arise such as missing data, biased data, imbalanced data. Researchers proposed an *enhanced whale optimization algorithm (EWOA) framework* [25] that combined the whale optimization algorithm (WOA) with the sine cosine algorithm (SCA) and logistic chaotic map (LCM) to achieve better accuracy and feature understanding. The original WOA had a strong exploration process but a weak exploitation process due to updating the position vector with a randomly chosen search agent instead of the optimal one. Additionally, the updating mechanism was performed randomly. To address these weaknesses, a Lévy flight mechanism was incorporated to ensure the equal use of two updating mechanisms.

Most studies focus on those neural network models, which are designed for a single course. However, while this approach may be accurate in some cases, it can be limiting when applied to other courses or predicting the overall performance of a student. Furthermore, overfitting can occur in the case of introducing new courses or the lack of course data can also trigger this result. Their findings [26] demonstrated the viability of their general, course-independent model created using *CatBoost* for risk assessment as early as two weeks into a course. CatBoost is a machine learning algorithm that uses gradient boosting to combine weak learners into a strong learner. It handles categorical values differently by randomly permuting the dataset and calculating the average label value for each object. Many researchers have used traditional machine learning algorithms when tackling this prediction problem. In the context of the educational area, little research has been carried out on the architecture of convolutional neural networks (CNNs). To predict academic success, they combined *two separate 2D CNN models and created a hybrid model* [27]. To evaluate the hybrid model’s performance, they converted the 1D sample data into 2D image data and then compared the model’s performance to various traditional baseline models. As we know, CNNs are mostly used for image detection, image classification, so this approach can be looked into the near future, because these models have been found to be promising in the field of computer vision (models such as you only look once (YOLO), RetinaNet).

### 2.5. Previous Applications of Support Vector Machines

Measuring and predicting students’ performance is a great way for educational institutes to improve the curriculum or the school’s studying atmosphere. In recent studies, among the machine learning algorithms, *support vector machine (SVM)* [28] has been shown to outperform other machine learning algorithms in terms of predicting student outcomes. In most studies, people generally opt to investigate with SVM techniques, which can be used extensively in classification problems. Results show [29] that using linear support vector machines can generate high accuracy values as well. The algorithm predicted that the parental level of education does not influence students’ performance. Features such as gender, race, and having regular lunches at school are considered impacting factors by the SVMs. In a recent study [8], researchers tried to compare random forests, nearest neighbor, support vector machines, logistic regression, naïve Bayes, and k-nearest neighbor algorithms using the final exam grades of undergraduate students. SVM and NN mostly outperformed all machine learning algorithms, with the decision tree showing the lowest performance, and thus should be abandoned. The studies discussed in the previous section highlight the importance of utilizing machine learning algorithms to predict and analyze student performance in educational settings. However, there is a need to establish stronger connections between the field of educational sciences and data science in order to fully address the issue of success in education. Further research should focus on developing models that take into account not only academic performance, but also social and emotional factors that impact student success. By establishing a more comprehensive understanding of the factors that contribute to student success, we can better inform educational policies and practices, ultimately leading to improved outcomes for students.

In this paper, our goal is to find the changes in student performance between online and offline data, and to assess whether the implementation of online learning was beneficial for the educational development of students. Thus, machine learning algorithms such as support vector machine, k-nearest neighbor, and decision tree have been implemented and examined. This paper can also show institutes why some students achieved worse results than others, which can be used as an indication for change in the education system.

The rest of this paper is arranged as follows. In Section 2, the previous work related to student performance prediction is introduced. Section 3 describes the two databases that were used in the machine learning algorithms. In Section 4, statistical hypothesis tests are made using the online and offline databases. Section 5 outlines the used machine learning algorithms and their limitations. Section 6 describes the methods used in the predictions, and in Section 7, conducted experiments and their outcomes are discussed. Section 8 describes discussions related to the results. The final conclusions are gathered in Section 9.1. Lastly, in Section 9.2, possibilities for future work are suggested.

## 3. Databases

Many databases had different numeric values, and the relationship between tables was complex, so we tried to find two databases that were simplified and that could give valuable information to this paper. Furthermore, most databases were about offline learning and many had only one or two subjects contained in one database, which we didn’t need. We found an offline database [30] from Kaggle called Higher Education Students Performance Evaluation, which was collected from the Faculty of Engineering and Faculty of Educational Sciences students in 2019. The purpose is to predict students’ end-of-term performances using machine learning techniques. The dataset contains the following categorical values, which are related to the students’ personal information like gender, parental status, or habits (these attributes are listed below in the hypothesis test). The dataset contains 33 attributes, and originally had 145 records.

**Figure 1:**
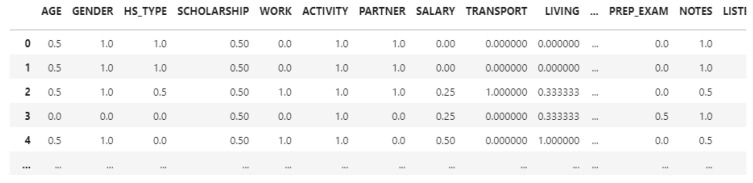
Offline after normalization.

For the prediction, we created a PASSED attribute, which is 0 if the OUTPUT Grade is 0, so the student failed in that semester, PASSED is 1 if the student got a non-failable final grade. We also deleted the ID of the table, because machine learning algorithms don’t need that information. Feature scaling was applied to the dataset, in order to maintain the values between 0 and 1, to achieve better training and testing results. After the normalization, the dataset looks like this (shown in Figure 1):

The dataset contains 33 attributes, and 145 records after the normalization and attribute selection (STUDENTID removed, PASSED added).

For the online dataset, we used the Student-Performance-Prediction-using-Data-Mining-Techniques github [31], where we found a data set of various undergraduate students which was compiled from March 2021. A Likert-type questionnaire was administered, and a large number of responses were gathered from various primary and secondary resources. With the help of a Google form, they could gather valuable information about students, which we could transform and use. Here, we also converted the categorical data to number classes and then we also used normalization. The dataset contains the following categorical values, which mostly state the student’s choice for each question in the survey (these attributes are listed below in the hypothesis test). The dataset contains 30 attributes, and 801 records originally.

**Figure 2:**
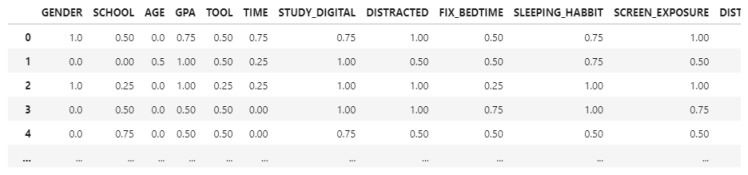
Online after normalization.

As we can see, the database consists of opinions about certain questions, such as DISTRACTED, meaning that a student was easily distracted during e-learning. Some attributes were removed because they contained unimportant values, such as before COVID-19 data, which is not needed in the online dataset. In addition, during the extract, transform, and load (ETL) process, rows containing at least one NaN value were removed. After the normalization, the dataset looks like this (shown in Figure 2):

The dataset contains 20 attributes, and 787 records after normalization and attribute selection (PASSED added, attributes related to pre-COVID-19 questions removed). Our main goal was to predict if a student will fail or pass with certain aspects such as studying hours, which was provided by the datasets.

## 4. Correlation Analysis—The Chi-Square Test

**Figure 3:**
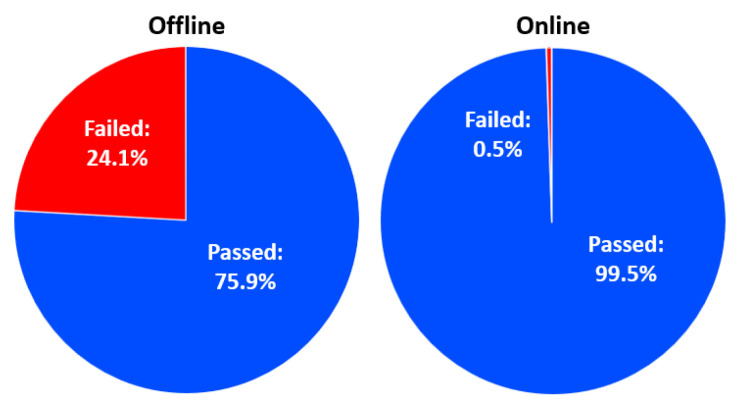
End-of-term performances: passing.

As we mentioned above, the main goal is to find the crucial factors that affect passing. Given that most of the students passed the exam, our goal is to decrease the student failure rate as much as possible. For visualization purposes, here are the passing ratios for each dataset (shown in Figure 3):

As we can see, the online database has one flaw: it has a very small number of people who did not pass. Later, we could conclude that this will cause balance issues in the SVM machine learning algorithm, but in other algorithms we got analyzable results.

In order to find the correlating attributes, we find a way to the associations between two attributes. The Cramer’s V correlation matrix was thus applied. When there is more than a 2×2 contingency, Cramer’s V is implemented to analyze the connection between two categorical variables. The link between a number of categorical variables is summarized in contingency tables, often known as crosstabs or two-way tables. It is a unique kind of frequency distribution table in which two variables are displayed at once. Cramer’s V ranges from 0 to 1 (inclusive). 0 means there is no relationship between the two variables. 1 denotes a significant correlation between the two variables. Cramer’s V can be calculated by using the below formula:

(1) $$\frac{\sqrt{\frac{X^{2}}{N}}}{min(C-1,R-1)}$$

Here,

$X^{2}$ is the Chi-square statistic;

N represents the total sample size;

R is equal to the number of rows;

C is equal to the number of columns.

**Figure 4:**
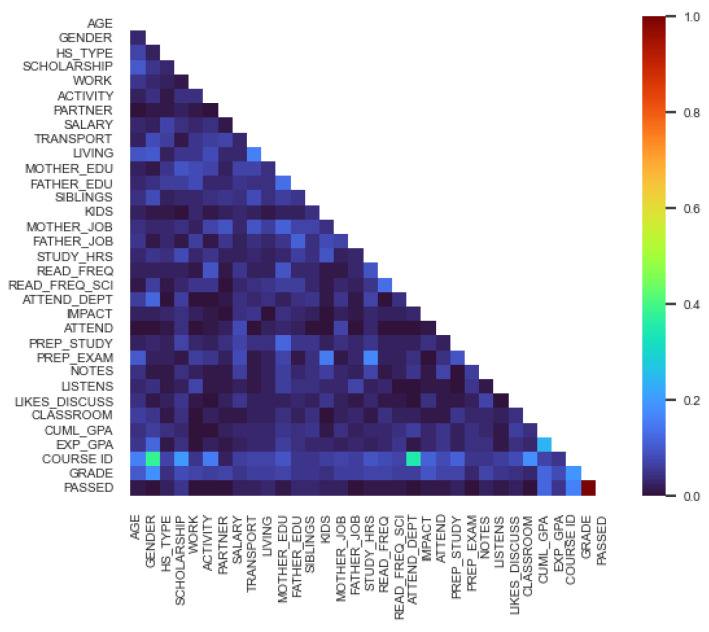
Cramer’s V matrix: offline.

With Cramer’s V, the following correlation matrix was the result offline data (shown in Figure 4):

We can conclude by the colors that the PASSED is in a relationship with HS_TYPE, TRANSPORT, SIBLINGS, MOTHERS_JOB, PREP_STUDY, CUML_GPA, COURSE_ID.

**Figure 5:**
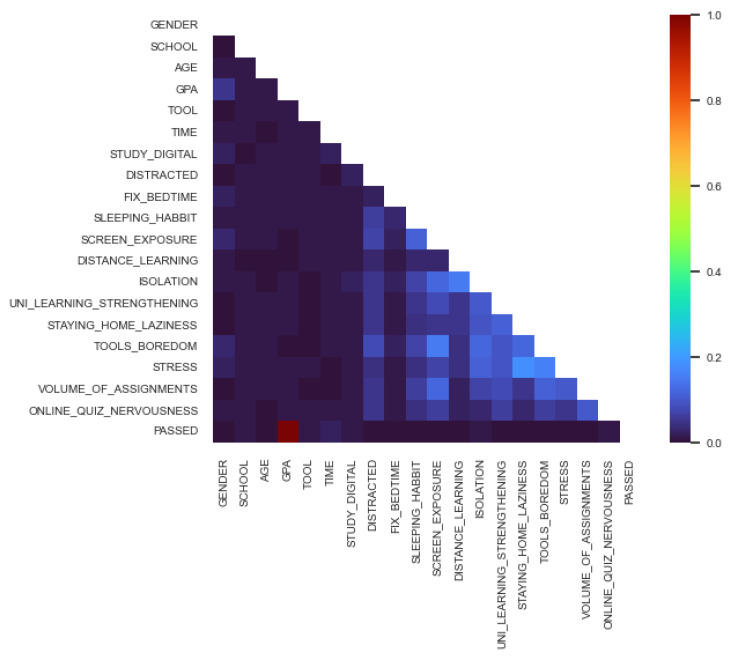
Cramer’s V matrix—Online.

With Cramer’s V, the following correlation matrix was the result for online data (shown in Figure 5):

We can conclude by the colors, that the PASSED is in relationship with GPA, TIME, SCHOOL, ISOLATION, STUDY_DIGITAL.

Are they really in relationship? The chi-squared significance test can be used to determine whether or not there is a correlation between them. The discrepancy between observed and expected frequencies of the results of a set of events or variables is measured by the chi-square statistic ($\chi^{2}$). Chi-square is helpful in examining these variations in category variables, particularly ones that are nominal in nature. The amount of dispersion between real and observed values, the number of degrees of freedom, and the sample size all affect the value of $\chi^{2}$. You can use $\chi^{2}$ to determine whether two variables are related or unrelated to one another. Additionally, it can be used to assess how well an actual distribution fits a hypothetical distribution of frequencies.

The formula for chi-square Is:

(2) $${\chi_{c}}^{2}=\sum \frac{{\left(O_{i}-E_{i}\right)}^{2}}{E_{i}}$$

Here,

c = Degrees of freedom;

O = Observed value(s);

E = Expected value(s).

A P value is calculated which tells us if they are in a strong or weak relationship. We set alpha=0.05 as a threshold index, which controls the result. With the values calculated on both datasets, let us find out if our null hypotheses are true.

We can see that with the hypotheses test shown in Table 1, we can conclude that the following attributes are in correlation with PASSED: CUML_GPA, COURSE ID, GRADE, GPA, TIME.

## 5. Used Machine Learning Algorithms

Machine learning (ML) is the method of gaining knowledge from inputs without requiring explicit programming by using algorithms and computational statistics. It belongs to the computer science branch of artificial intelligence. The following algorithms were tested for the prediction of student performances, and for detecting factors that affect low grade quality:

### 5.1. Support Vector Machine

The Support Vector Machine (SVM) [32] is a supervised machine learning algorithm. It is commonly used in the fields of data science and machine learning, because it is extremely strong and adaptable. Its main feature is that it can use linear or nonlinear methods for solving classification tasks; it is also used for regressions or finding outliers in a large dataset. In data mining it is generally used among small- or medium-sized databases, where a classification is due to happen. In order to distinguish between the many classes in the dataset, the support vector machine is a non-probabilistic linear classifier.

**Figure 6:**
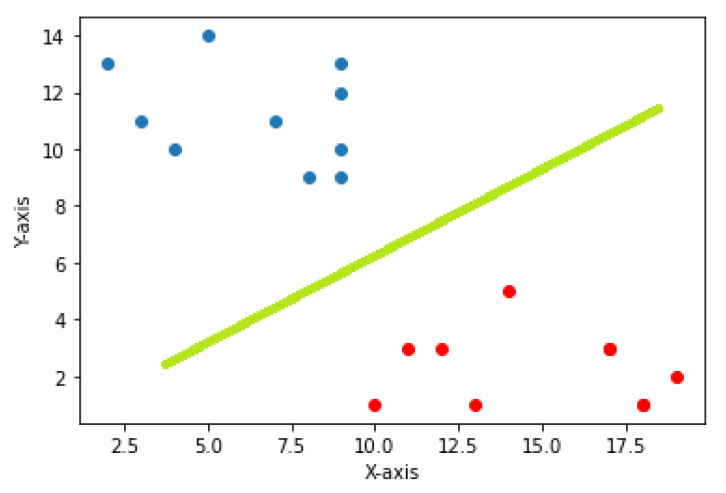
Support vector machine hyperplane [33].

The data points on the n-dimensional graph are called vectors. The support vectors are mainly closer to the hyperplanes and affect the orientation of the hyperplane (shown in Figure 6). We pass both the positive and negative edges of the hyperplane through this support vector.

In an *n*-dimensional dataset, the hyperplane sets a decision boundary with (n−1) dimensions, used to separate the different classes in a classification problem.

In order to transform non-linear data into higher dimensions, a mathematical function known as the kernel is used in the SVM. The kernel helps the SVM to separate different classes by using hyperplanes. There are multiple types of kernels, which will be further discussed in our prediction results, such as linear, poly, rbf, or sigmoid. Additionally, with the help of programming languages such as Python, people can also create their own kernels.

**Figure 7:**
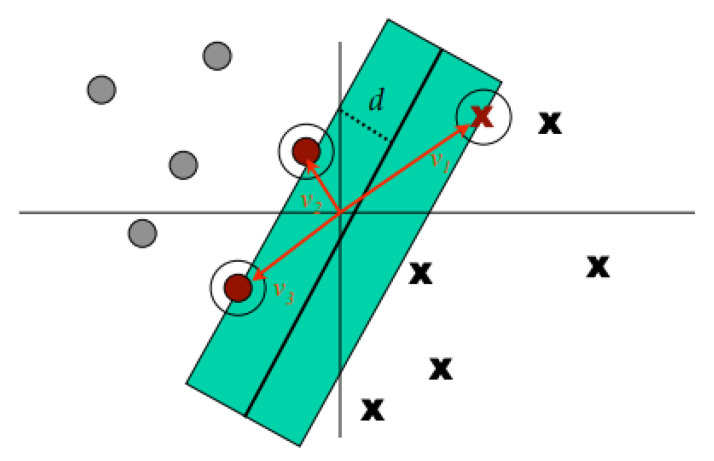
Support vector machine margin [34].

In a classification task, the job of SVMs is to maximize the separation between the hyperplanes in order to obtain optimal hyperplanes. By achieving the correct separation, a classifier with a large margin can be obtained (shown in Figure 7), resulting in points in the plane being well-separated and correctly classified, thus achieving high accuracy results.

### 5.2. K-Nearest Neighbor

K-nearest neighbors (K-NN) [35] is a supervised learning algorithm for both regression and classification. It attempts to predict the correct class of the test data by computing the distance between the test data and the training points. The algorithm then chooses the K-score that best matches the test data. For classification, the ANN algorithm calculates the probability that the test data belongs to the “K” class of the training data, which contains the highest probability. For regression, the value selected is the mean of the “K” training points.

The purpose of the K-NN algorithm is to classify a new data point, $x_{1}$, into either class A or class B. This type of problem can be solved by employing the K-NN algorithm, which can easily identify the class or category of a particular record.

**Figure 8:**
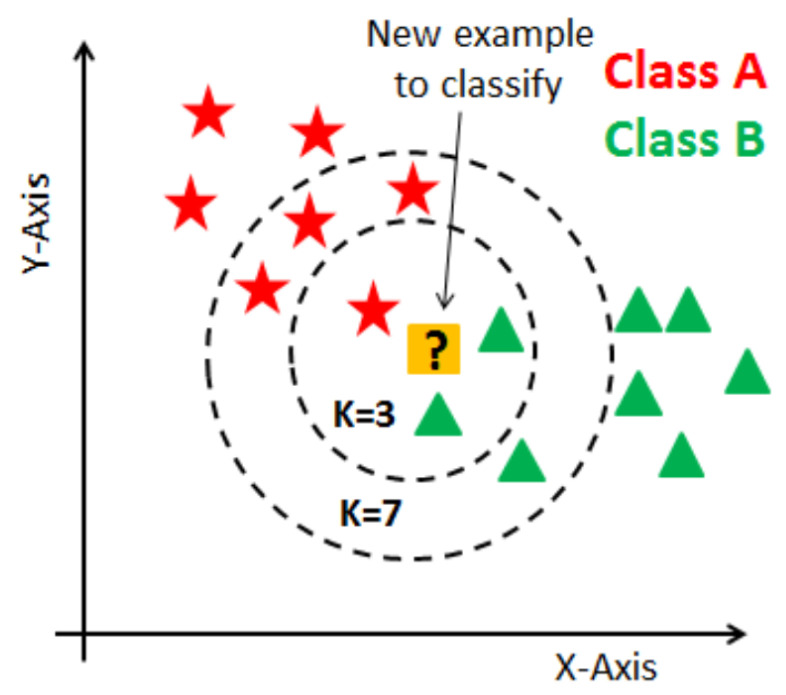
K-nearest neighbor algorithm [36].

The algorithm works the following way (shown in Figure 8):

When starting the algorithm, the user can either select the K number or it can be estimated. K is the number of neighbors used for calculating the distances. After that, the algorithm takes the K nearest neighbors according to the distance calculation method (Euclidean, Manhattan, or Hamming). It then determines the quantity of data points in each category among these K nearest neighbors. Finally, it allocates the new data points to the class for which the number of neighbors is maximum. The distance between the new point and each training point can be calculated using various algorithms. Examples of such algorithms include Euclidean distance, Manhattan distance, and Hamming distance:

1. Euclidean distance (for continuous): Euclidean distance is calculated as the square root of the sum of the squared differences between points.
2. Manhattan distance (for continuous): This is the distance between vectors using the sum of their absolute difference.
3. Hamming distance (for categorical): It is used for categorical variables. If the *x* and the *y* are equal, the distance D will be equal to 0. Otherwise, the distance is 1.

K indicates the number of nearest neighbors. The distances between the test points and the trained label points must be calculated iteratively, as new points are added simultaneously and the distance metrics must also be updated. When initializing the algorithm, the K value can be chosen randomly, then making calculations iteratively to find the perfect K value. A small K value can make for an unstable decision boundary, so a significant value of K is advisable for classification, leading to a smoother decision boundary.

### 5.3. Decision Tree

The supervised learning algorithm family includes the decision tree (DT) algorithm [37]. The decision tree approach can be used to resolve both classification and regression challenges, unlike other supervised learning techniques. By learning straightforward decision rules from prior (training) data, a decision tree can be used to build a training model that enables one to predict the class or value of the target variable.

Decision trees can be used for both categorical and continuous variables. When splitting the tree, the homogeneity of the resulting subnodes increases, meaning that the nodes start to belong to a similar class. For categorical variables, the decision strategy for splits involves evaluating the purity of the resulting nodes according to the class labels. For continuous variables, the decision strategy involves evaluating the homogeneity of data values in relation to the splitting criterion.

There are various algorithms used in decision trees, such as ID3, C4.5, CART, CHAID, and MARS. The decision tree divides nodes into all potential variables before selecting the split that results in the subnodes with the highest homogeneity. Choosing an algorithm depends on the kind of target variable.

Researchers developed solutions for addressing this attribute selection problem. They recommended utilizing criteria such as entropy, information gain, Gini index, gain ratio, reduction in variance, and chi-square. For classification or attribute selection, researchers worked on creating criteria such as entropy, information gain, Gini index, gain ratio, reduction in variance, and chi-square. These criteria generate values for every attribute. Then, the tree nodes are sorted by the criteria, and the attribute with the highest value is placed at the root node. When using information gain, the data should be categorical, and for the Gini index, it should be continuous.

### 5.4. Random Forest

A random forest [38] is a machine learning technique used to solve regression and classification problems by combining multiple classifiers with cooperative learning. It is called a "forest" because it consists of many decision trees. Bagging or bootstrap aggregation is used to generate the random forest, with bagging being an ensemble meta-algorithm that increases precision. Predictions are made in multiple decision trees, and the result is chosen based on their outputs. The accuracy of the result is increased by increasing the number of trees, though this also increases resource needs. The random forest removes the limitations of a decision tree algorithm by reducing overfitting of the records and increasing the accuracy of the models.

The decision tree algorithm is outperformed by this one in terms of accuracy. It can produce reasonable predictions and manage missing data without tuning hyperparameters. At the node-split point in every random forest tree, a subset of features is chosen at random.

**Figure 9:**
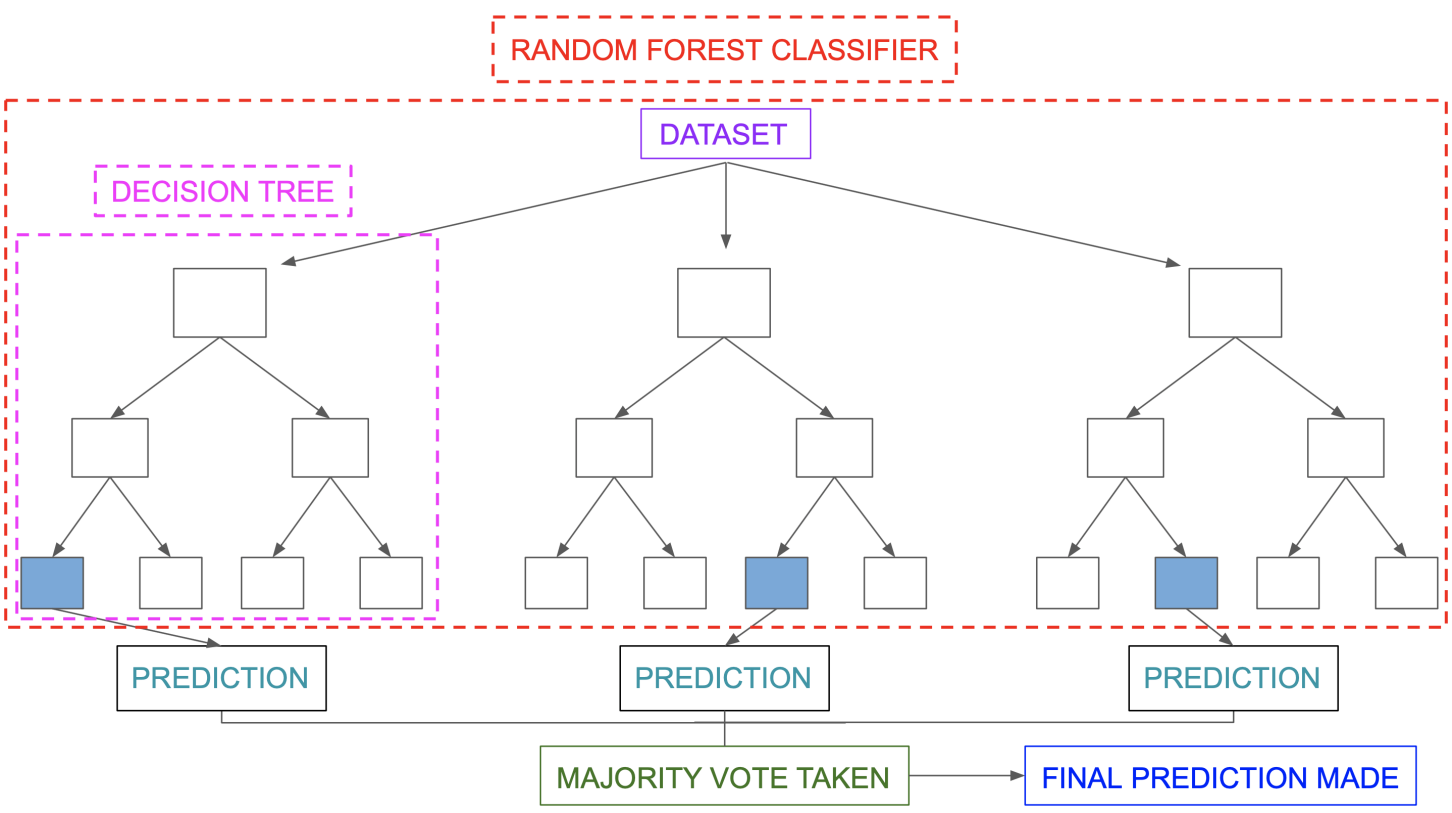
Random forest algorithm [39].

The construction of root nodes and the separation of nodes necessary for the random forest algorithm, in contrast to the decision tree algorithm, require prediction through bagging. Bagging involves using different samples instead of just one sample, and predictions are made using features and observations from a training dataset. Depending on the training data that is provided to the random forest algorithm, decision trees produce various results (shown in Figure 9).

### 5.5. Limitations of the Different Algorithms and Databases

*SVM* requires significant computational resources for large datasets and can overfit if the kernel function or regularization parameter is not chosen carefully. It is sensitive to noise and can be biased towards the majority class in imbalanced datasets. It is a binary classification algorithm and requires modifications for multi-class classification problems.

*K-NN* has no training time, but its time complexity increases with the number of training samples. It is sensitive to noisy data and outliers, and its performance depends on the optimal value of K. KNN becomes less effective with an increase in the number of features, known as the curse of dimensionality.

*Decision trees* can be prone to overfitting, can be unstable and biased towards certain features, and may not capture complex relationships. They are built using a greedy approach which may not consider future splits, leading to suboptimal trees. Furthermore, decision trees are not robust to noise or outliers and may overemphasize irrelevant features.

*Random forest* can be difficult to interpret, can overfit, and may be biased towards certain features. It is computationally expensive, not suitable for extrapolation tasks, and may not perform well with certain types of features or imbalanced datasets.

The online database lacks data on failed students, which limits the effectiveness of the trained models.

## 6. Method

In this section, the proposed algorithms, steps of getting the results, and the chosen environment are presented. We chose Jupyter Notebook as our environment, as it is suitable for our problem. Jupyter Notebook is an open-source web application that allows users to create and share documents containing live code, equations, visualizations, and narrative text. It supports over 40 programming languages, including Python, which makes it ideal for data science, scientific computing, and machine learning.

The algorithms were implemented using the Python language, and the following libraries were used in order to solve and visualize the problem:

- numpy: a library for numerical computing in Python;
- pandas: a library for data manipulation and analysis;
- seaborn: a library for data visualization based on matplotlib;
- matplotlib: a library for creating static, animated, and interactive visualizations in Python;
- time: a library providing various time-related functions;
- sklearn: a machine learning library for Python;
- astropy: a library for astronomy and astrophysics;
- scipy: a library for scientific and technical computing;
- scikit-learn: a library for machine learning algorithms and tools in Python.

Before implementing the algorithm, the ETL process was carried out to ensure that the dataset was in the correct format and that the most suitable attributes were selected. The datasets were in a .csv format, which was read, and the feature selection was made (selected attributes are listed above in the Dataset Section).

Normalization was used because it can improve the performance and training of machine learning models. When the features have different scales, some features may have more influence on the model than others, leading to inaccurate predictions. Normalization helps to eliminate this problem by giving equal importance to all features. Additionally, normalization can improve the convergence speed of some optimization algorithms used in machine learning. In our case, the min-max normalization technique was selected, which is a data preprocessing technique used to rescale numeric data to a specific range, typically between 0 and 1. It involves subtracting the minimum value of the data and dividing the result by the range of the data. This technique is useful for preventing some features from dominating others in machine learning models.

After normalization, the four machine learning algorithms were implemented in the following ways:

- Support vector machine (SVM) was used with three different kernels (linear, polynomial, Gaussian). The algorithm splits the data into training, validation, and testing sets, and tunes hyperparameters to find the optimal C value for the SVM algorithm. Then, the SVM model is trained with the optimal C value and the testing set is used to evaluate the model’s accuracy and F1 score. This process is repeated until a certain threshold of accuracy and F1 score is reached. Finally, the results are displayed. The max_iteration limit was set to 100, as it gave the best result for that value. The optimal split states for the linear kernel, polynomial kernel, and Gaussian kernel were 388,628,375; 7,070,621 and 93,895,097; respectively. Random splitting of the data was used to avoid overfitting and to get a generalizable model. By setting the seed to a specific number, we can ensure that the data is split in the same way each time the code is run.
- The k-nearest neighbors (KNN) algorithm was used to classify a dataset into two classes (binary classification). The dataset was loaded into *x* and *y*, where *x* contained the features and *y* contained the target variable (the class labels). To find the optimal random state, the dataset was randomly split into training and testing sets, and a KNN model was trained. Its performance was then evaluated using accuracy and F1 score. The optimal state was chosen based on the highest accuracy and F1 score obtained. Once the optimal state was found, the dataset was split again, and a new KNN model was trained on the training set with fixed hyperparameters (n_neighbors = 7, metric = ’chebyshev’). GridSearchCV was used to determine the best hyperparameter for the number of neighbors; however, it was not used to train the final KNN model. 7 was chosen as the K value, because after tuning the parameters 7 or 11 was the optimal candidate.
- This decision tree classifier was used to classify a dataset into two classes (binary classification). The dataset is loaded into *X_1* and *y_1*, where *X_1* contains the features and *y_1* contains the target variable (the class labels). The algorithm performs a train-test split and fits a decision tree classifier on the training set. The algorithm also performs cross-validation using both the k-fold and stratified k-fold methods, with both 5- and 10-folds, to evaluate the performance of the model. The macro-averaged scores for accuracy, precision, recall, and F1-score are computed using the *cross_validate* function.
- The algorithm trains a random forest classifier model to predict whether a student has passed a course or not, based on various features related to their demographics, academic performance, and personal habits. The model is evaluated using a train-test split, as well as cross-validation via both k-fold and stratified k-fold methods. Metrics such as accuracy, precision, recall, and F1 score are used to evaluate the model’s performance. Cross-validation is also performed using the k-fold and stratified k-fold methods to evaluate the model’s performance.

## 7. Results

**Figure 10:**
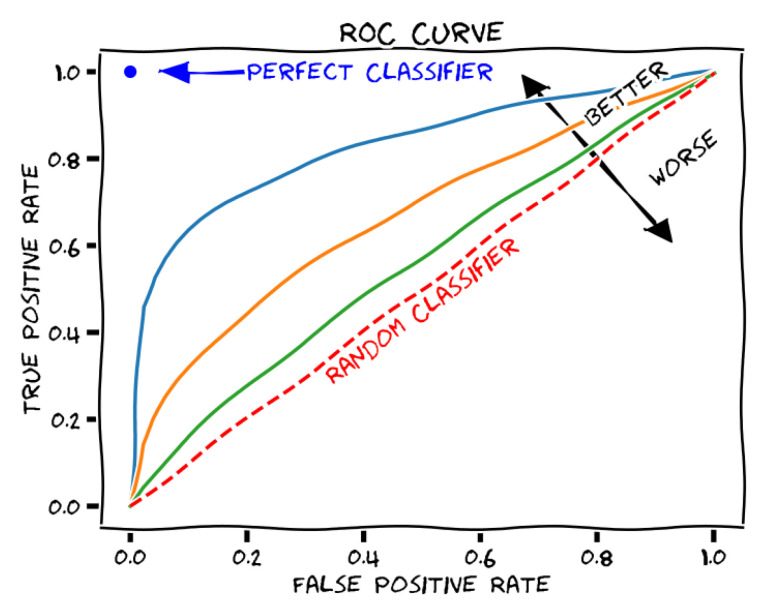
ROC curve description [40].

For better understanding the training results, first, we are going to introduce the receiver operating characteristic (ROC) curve (shown in Figure 10). A receiver operating characteristic (ROC) curve is a graphical representation of how a binary classifier system’s diagnostic capacity changes as the discrimination threshold is altered. The area under the ROC curve, also known as the ROC score, is used to measure the performance of the classifier system. The optimal ROC score is 1, which signifies a perfect classifier system.

The excellence of the model can be declared by calculating the F1 score and the accuracy. The F-measure, often referred to as the F1-score, is a measure of a test’s accuracy used in statistical analyses of binary categorization. It is derived from the test’s precision and recall, where precision is the ratio of true positive results to all positive results, including those incorrectly identified as positive, and recall is the ratio of the number of true positives to the total number of samples that should have been classified as positive.

**Figure 11:**
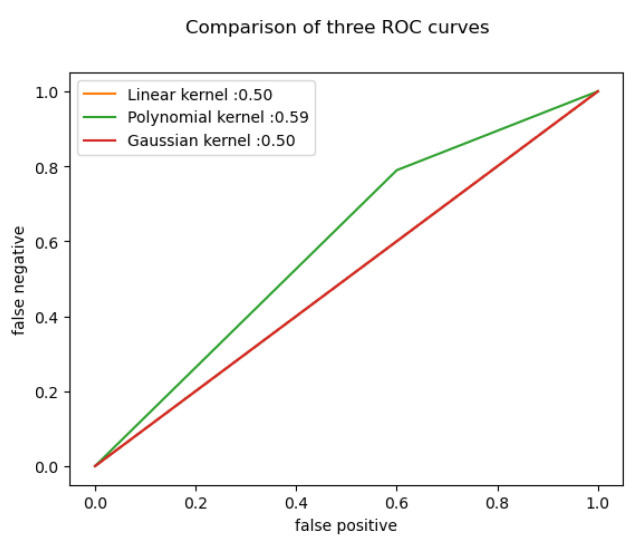
Offline ROC curve.

The results are the following for the different algorithms (shown in Figure 11 as well):

### 7.1. Support Vector Machine

For SVM we used the following kernels:

1. Linear kernel: When the data can be divided using a single line, or when it is linearly separable, a linear kernel is utilized. It is usually applied when a particular dataset contains a sizable number of features.
2. Polynomial kernel: A kernel function called a polynomial kernel, which is frequently used with SVMs and other kernelized models, shows how similar vectors in a feature space are to the polynomials of the original variables and enables the learning of non-linear models.
3. Furthermore, Gaussian radial basis function (RBF) is a well-liked kernel approach used in SVM models. The value of an RBF kernel relies on how far it is from the origin or another location.

For the offline dataset, the linear and the Gaussian kernels were the exact same in performance, accuracy and F1 score. Therefore it was easily distinguishable which kernel did the best during the training. In addition, for every training and testing, the dataset splitting was 80–20 for all cases.

By calculating the SVM parameters, we can identify the maximum and minimum coefficients, enabling us to determine the most significant factors affecting students’ academic performance. The results presented in Table 2 indicate that the linear kernel SVM outperformed the polynomial and Gaussian kernel SVMs in terms of accuracy and misclassification rates. However, the polynomial kernel SVM achieved higher F1 and ROC_AUC scores, suggesting that it may be more appropriate for certain applications. Therefore, the choice of SVM kernel should depend on the specific use case and the cost associated with false negatives. Further experimentation and analysis are necessary to determine the optimal model for particular applications. Overall, the SVM classifier demonstrates promising results for offline dataset classification.

Factors that contribute to students’ success include EXP_GPA, PREP_STUDY, PARENTAL_STATUS, and GRADE, while ACTIVITY, PARTNER, NOTES, and ATTEND are factors that contribute to students’ failure.

**Figure 12:**
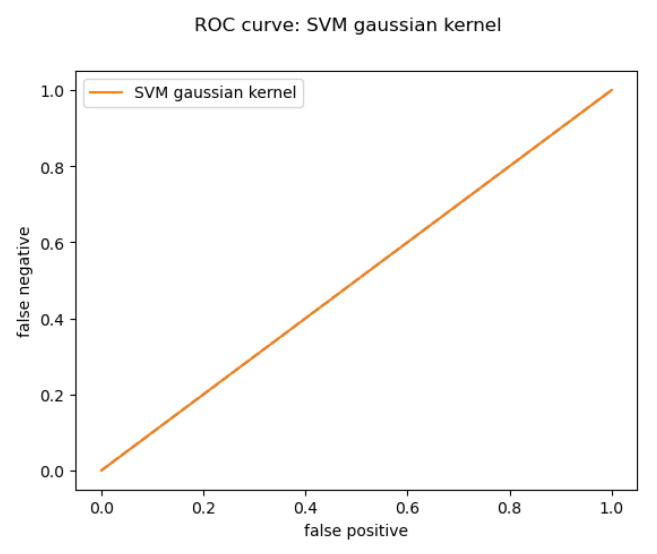
Online Gaussian kernel ROC curve.

For the Online dataset, only the Gaussian kernel worked, because the dataset has some balance issues, which can be solved by collecting more data about the not passing students. The result for the Gaussian kernel (shown in Figure 12 and Table 3):

By calculating the SVM parameters, we can sort them into max and min coefficients; this way we can get the major factors for the students’ performance issues.Factors helping students succeed: ONLINE_QUIZ_NERVOUSNESS, SCHOOL, FIX_BEDTIME, GPA. Factors leading students to failure SCREEN_EXPOSURE, SLEEPING_HABBIT, TIME, GENDER.

In the context of the student performance dataset, this means that the SVM model is able to predict with high accuracy whether a student will pass or fail based on their demographic, socioeconomic, and academic information.

### 7.2. K-Nearest Neighbors

In most trainings the ROC curve was below the optimal line. So we needed to find the best number of neighbors in order to achieve better results. In the knn_cv class in python there is a variable (knn_cv.best_params_), which can give the best K parameter, but before that some calculation needs to be carried out (e.g., GridSearch, fitting, finding the best score). With this analogy, we could find for the offline dataset 7 or 11 as values for K and for the online dataset 3 was the best option. With these values, the classification reports are shown in Table 4.

As we can see, the KNN algorithm performed better on the online dataset compared to the offline dataset. However, it is worth investigating if the algorithm’s superior performance on the online dataset is due to the lack of patterns in the dataset (Future Work).

For the Offline evaluation, the precision, recall, and F1-score of the two classes (0 and 1) are displayed, along with their corresponding support (number of instances in each class) and the overall accuracy. The precision, recall, and F1-score for class 0 are all zero, indicating that the model was unable to correctly identify any instances of class 0 in the offline evaluation. However, the precision, recall, and F1-score for class 1 are 0.70, 1.00, and 0.83, respectively, indicating that the model achieved good performance in correctly identifying instances of class 1. The overall accuracy for the offline evaluation is 0.70.

For the online evaluation, the precision, recall, and F1-score for both classes (0 and 1) are displayed, along with their corresponding support (number of instances in each class) and the overall accuracy. The precision, recall, and F1-score for both classes are perfect (1.00), indicating that the model achieved perfect performance in correctly identifying instances of both classes in the online evaluation. The overall accuracy for the online evaluation is 1.00.

The offline evaluation showed poor performance in correctly identifying instances of class 0, while the online evaluation showed perfect performance in correctly identifying instances of both classes. It is worth mentioning that the KNN algorithm outperformed the SVM algorithm in terms of accuracy scores, thanks to the optimal k selection.

KNN is a non-parametric method that does not make any assumptions about the distribution of the data, making it a good choice when there is no prior knowledge about the data. In the context of the student performance dataset, KNN can classify students as pass or fail based on their similarity to other students in the dataset.

### 7.3. Decision Tree

The results of the decision tree algorithm were quite interesting. We used k-fold and stratified k-fold cross validation (with 5 and 10 folds) to evaluate the algorithm. The results presented in Table 5 show that the decision tree (DT) model achieved perfect accuracy of 100% on both the offline and online datasets. The precision, recall, and F1-score for each class were also 1.00, indicating that the model correctly classified all samples. The confusion matrices further confirm the perfect performance of the DT model, with no misclassified samples in either dataset. However, it is important to note that the perfect performance may be due to overfitting to the specific dataset used in this study. Further experimentation and analysis may be necessary to determine the generalizability of the model. In conclusion, the DT model appears to be a highly effective model for classifying the given datasets.

### 7.4. Random Forest

As for the random forest algorithm, the results were quite interesting. We evaluated the model using k-fold and stratified k-fold cross-validation (CV) with 5 and 10 folds, and we found that it gave the best accuracy results for the online dataset. The results in Table 6 show that the random forest classifier performed well on both the offline and online datasets, achieving an accuracy of 0.95 and 1.00, respectively. The model had high precision and recall for class 1 in both datasets, with a precision of 1.00 and a recall of 0.94 for the offline dataset, and a precision and recall of 1.00 for the online dataset. However, the precision for class 0 in the offline dataset was lower at 0.80, which led to some misclassifications. The F1 scores for the model were high for both datasets, with a weighted F1 score of 0.96 for the offline dataset and 0.99 for the online dataset. Overall, these results suggest that the random forest classifier is a strong performer for this classification task. However, further experimentation and analysis are necessary to determine the generalizability of the model.

## 8. Discussion

The classification results for both offline and online student performance prediction using random forest (RF) algorithm showed promising outcomes. The offline prediction achieved an accuracy of 95% with a weighted F1-score of 0.96, while the online prediction achieved perfect accuracy of 100% with a weighted F1-score of 0.99. The RF algorithm appeared to be highly effective in predicting student performance using the given features in both offline and online settings. The algorithm was able to achieve high precision, recall, and F1-score for both the classes, indicating that it can accurately identify students who are likely to perform well and those who may struggle.

However, the results also revealed that the performance of the algorithm varied significantly between offline and online settings. While the offline prediction had a relatively smaller dataset with only 44 samples, the online prediction had a much larger dataset with 237 samples. The results suggest that the RF algorithm may require a larger dataset to improve its performance in offline settings. Further investigation is required to determine whether the discrepancy in performance is due to the difference in dataset size or some other factor. Overall, the RF algorithm can be considered as a promising approach for predicting student performance in both online and offline settings.

## 9. Conclusions and Future Work

### 9.1. Conclusions

Dropout is a major concern in online education, and online education platforms still struggle with high dropout rates and accurately predicting and reducing them. Through our research, we have concluded that the main factors affecting a student’s performance are habits, such as sleeping, studying hours, and note-taking. Additionally, relationships, such as the “partner” attribute, can also impact performance, as noted in the factors leading to failure in the SVM algorithm. The resulting attributes indicate that the hypothesis test was reasonably accurate for both the online and offline databases. For the online database, studying time, GPA, and school type appeared in both results, while for the offline database, achieved grade, course type, and family-related attributes appeared in both results, supporting the original hypothesis statements.

For the two datasets, the decision tree algorithm achieved the best accuracy results for the offline dataset, while the random forest algorithm performed best for the online dataset. These results could be further analyzed using a wider variety of databases.

Overall, the application of ML algorithms in education has the potential to improve academic performance by enabling personalized learning, adaptive testing, and data-driven decision-making. However, it is important to note that the success of these approaches depends on the quality of the data used and the accuracy of the algorithms employed.

### 9.2. Future Work and Suggestions

In terms of future work, several areas of correlation detection were not explored, such as multivariable correlations, which could provide more detailed explanations for the poor performance of students. Additionally, new datasets can be collected to address the issues of unbalanced data in SVM algorithms. As seen in the results, decision tree and random forest algorithms appear to be effective in predicting student performance, and further analysis of these models is warranted.

It is important to consider tuning parameters such as the maximum depth of the tree, the minimum number of samples required to split an internal node, and the minimum number of samples required to be at a leaf node, as these can significantly improve model accuracy. Random forest is an ensemble learning method that combines multiple decision trees to improve model accuracy, and techniques such as bagging, boosting, and stacking can further enhance performance. Decision trees and random forests can handle missing values, but the method used to handle missing values can affect model accuracy. One approach is to use imputation techniques to fill in missing values, while another is to treat missing values as a separate category. Simplifying the tree by removing branches that do not improve model accuracy can also enhance generalization performance and prevent overfitting.

## Figures and Tables

**Table 1:** Chi-squared test results.

Online			Offline		
**Attribute**	**P**	**In Relationship with PASSED**	**Attribute**	**P**	**In Relationship with PASSED**
GENDER	1.0		1- Student Age	0.5653471007309754	
SCHOOL	0.30312215312723617		2- Sex	0.5523722889801679	
AGE	0.2953693982712599		3- Graduated high-school type	0.24781084162944328	
GPA	$3.0519065109580407\times 10^{-169}$	✓	4- Scholarship type	0.11572254970006551	
TOOL	0.28673716710442554		5- Additional work	0.7826380994749932	
TIME	0.008962416672052801	✓	6- Regular artistic or sports activity	0.5523722889801679	
STUDY_DIGITAL	0.08621366366725645		7- Do you have a partner	0.6303571835687162	
DISTRACTED	0.7399025825207426		8- Total salary if available	0.6099332258713447	
FIX_BEDTIME	0.6908995564635236		9- Transportation to the university	0.5322122277880168	
SLEEPING_HABBIT	0.47716005566088127		10- Accommodation type in Cyprus	0.20099557574466903	
SCREEN_EXPOSURE	0.9837331924869825		11- Mother’s education	0.8447430862982488	
DISTANCE_LEARNING	0.87106656348976		12- Father’s education	0.4446173066557606	
ISOLATION	0.19302015313488863		13- Number of sisters/brothers if available	0.3751796318164345	
UNI_LEARNING_STRENGTHENING	0.7302432920209609		14- Parental status	0.26226459029998406	
STAYING_HOME_LAZINESS	0.9494226741965801		15- Mother’s occupation	0.5866029226108516	
TOOLS_BOREDOM	0.857639492044402		16- Father’s occupation	0.970296826082659	
STRESS	0.9438510256976413		17- Weekly study hours	0.730484272332087	
VOLUME_OF_ASSIGNMENTS	0.6252988719785977		18- Reading frequency non-scientific books/journals	0.6578769407759745	
ONLINE_QUIZ_NERVOUSNESS	0.2998717029054319		19- Reading frequency scientific books/journals	0.47322234454747136	
			20- Attendance to the seminars/conferences	0.6301485020030153	
			21- Impact of your projects/activities on your success	0.36687804454852474	
			22- Attendance to classes	0.16635800926992822	
			23- Preparation to midterm exams 1	0.31046666478253787	
			24- Preparation to midterm exams 2	0.7229766411774667	
			25- Taking notes in classes	0.3169771850067032	
			26- Listening in classes	0.670788988176968	
			27- Discussion improves my interest	0.31097735731866283	
			28- Flip-classroom	0.7258411373761349	
			29- Cumulative grade point last semester (/4.00)	0.001270588208597507	✓
			30- Cumulative grade point in the graduation (/4.00)	0.06807720013490783	
			31- Course ID	0.0026331340604132353	✓
			32- OUTPUT Grade	$4.549289632793446\times 10^{-28}$	✓

**Table 2:** Offline Dataset SVM.

Metric	Linear Kernel	Polynomial Kernel	Gaussian Kernel
**Training time**	1 ms	0 ms	1 ms
**Accuracy %**	87.5	70.8	70.83
**Confusion matrix**	[0, 7]	[2, 3]	[0, 3]
	[0, 17]	[4, 15]	[0, 21]
**F1 score**	0.41	0.59	0.47
**ROC_AUC_score**	0.50	0.59	0.50

**Table 3:** Online Dataset SVM.

The model accuracy: 98%
The training time is: 0 ms
The F1 score is: 0.5
The ROC_AUC_score is: 0.5

**Table 4:** KNN Classification Report.

Offline					Online				
	**Precision**	**Recall**	**F1-Score**	**Support**		**Precision**	**Recall**	**F1-Score**	**Support**
**0.0**	0.00	0.00	0.00	13	**0.0**	0.00	0.00	0.00	1
**1.0**	0.70	1.00	0.83	31	**1.0**	1.00	1.00	1.00	236
**Accuracy**			0.70	44	**Accuracy**			1.00	237
**Macro avg**	0.35	0.50	0.41	44	**Macro avg**	0.50	0.50	0.50	237
**Weighted avg**	0.5	0.7	0.58	44	**Weighted avg**	0.99	1.00	0.99	237

**Table 5:** DT Classification Report.

Offline					Online				
	**Precision**	**Recall**	**F1-Score**	**Support**		**Precision**	**Recall**	**F1-Score**	**Support**
**0.0**	1.00	1.00	1.00	8	**0.0**	1.00	1.00	1.00	1
**1.0**	1.0	1.00	1.00	36	**36**	1.00	1.00	1.00	236
**Accuracy**			1.00	44	**Accuracy**			1.00	237
**Macro avg**	1.00	1.00	1.00	44	**Macro avg**	1.00	1.00	1.00	237
**Weighted avg**	1.00	1.00	1.00	44	**Weighted avg**	1.00	1.00	1.00	237
**Confusion matrix** **[[8, 0]** **[0, 26]]**					**Confusion matrix** **[[1, 0]** **[0, 236]]**				

**Table 6:** RF classification report.

Offline					Online				
	**Precision**	**Recall**	**F1-Score**	**Support**		**Precision**	**Recall**	**F1-Score**	**Support**
**0.0**	0.80	1.00	0.89	8	**0.0**	0.00	0.00	0.00	1
**1.0**	1.00	0.94	0.97	36	**36**	1.00	1.00	1.00	236
**Accuracy**			0.95	44	**Accuracy**			1.00	237
**Macro avg**	0.90	0.97	0.93	44	**Macro avg**	0.50	0.50	0.50	237
**Weighted avg**	0.96	0.95	0.96	44	**Weighted avg**	0.99	1.00	0.99	237
**Confusion matrix** **[[8, 0]** **[2, 34]]**					**Confusion matrix** **[[0, 1]** **[0, 236]]**				

## Data Availability

The data presented in this study are openly available at https://www.kaggle.com/datasets/csafrit2/higher-education-students-performance-evaluation (accessed on 1 November 2022) and at https://github.com/kartikaya924/Student-Performance-Prediction-using-Data-Mining-Techniques (accessed on 1 November 2022).
